# Structural colors of pearls

**DOI:** 10.1038/s41598-021-94737-w

**Published:** 2021-07-27

**Authors:** Ryotaro Ozaki, Kei Kikumoto, Masataka Takagaki, Kazunori Kadowaki, Kazushi Odawara

**Affiliations:** 1 Department of Electrical and Electronic Engineering and Computer Science, Graduate School of Science and Engineering, Ehime University, Matsuyama, 790-8577 Japan; 2 Fisheries Research Center, Ehime Research Institute of Agriculture, Forestry and Fisheries, 5516 Shimonada, Uwajima, Ehime 798-0104 Japan

**Keywords:** Biomineralization, Photonic crystals

## Abstract

The luster is the most important characteristic of pearls, whose colors depend on periodic structures of aragonite crystal layers and conchiolin sheets. We here propose an optical model for analyzing the structural colors of pearls that includes the transmission, reflection, and scattering of light in pearls. Unlike other structural color materials, internal light scattering and its transmission are the keys to understanding the optical properties of pearls. The appearance of pearls is determined by the superposition of transmitted and reflected light. The transmission and reflection spectra of pearls calculated using the proposed model show good agreement with experimental results. We also demonstrate the rendering of images of pearls using the calculated spectra. Furthermore, the appearance of pearls with different layer thicknesses are predicted by calculation based on the optical model.

## Introduction

Pearls are gifts of nature and one of the most fascinating gems. Most gems such as gold, diamonds, sapphires, and rubies are mined from the ground, but pearls are produced by mollusk shells that live in seas or lakes. Among these gems, pearls are the only gems that humans can create from nature. The luster and color of pearls are known as structural colors, which originate from multiple reflections in a nanolayered structure of nacre (also called mother of pearl). Nacre is composed of aragonite crystal layers separated by conchiolin, which is a protein secreted by mollusk shells. The thickness of these layers determines the color of reflection from the nacre. To identify the cause of iridescence of pearls and shells, many researchers have studied the optics of pearls. Historically, Brewster studied diffraction at steps on a surface of mother of pearl from an optical viewpoint in his book published in 1833^1^. In 1917, Pfund measured the reflection spectra of mother of pearl in the visible and infrared regions^2^. He was the first to demonstrate that the interference color of mother of pearl is caused by multiple reflections from the multilayered structure of nacre. Rayleigh theoretically studied the iridescent color of mother of pearl around the same time^3^. In the 1930s and 1950s, Raman studied in detail the reflection, transmission, and scattering of light in pearls and shells to investigate the appearance of natural and cultured pearls^4–9^. Optical properties of pearls and shells have been studied by physicists in the past, but recently, genetic and DNA studies have been actively conducted^10–13^.

Structural colors are abundant in nature, for example, in pearls, opals, morpho butterflies, jewel beetles, and pollia fruits^14–20^. Optical calculations of structural colors are important both physically and practically. In 1917, Rayleigh theoretically calculated reflections from multilayered structures to study structural colors of some beetles and butterflies having a regularly stratified medium^14^. In modern optics, many optical elements and devices have been developed on the basis of thin-film interferences or multiple reflections in periodic structures, e.g., gratings, Fabry–Perot structures, and photonic crystals^21,22^. These optical elements have been used in spectrometers, lasers, optical filters, and other devices. The key features of these elements are wavelength selectivity and field enhancements^23,24^. Visualizations of structural colors have also been developed with the advancement of theoretical and numerical calculations for these modern optical elements. The realistic rendering of structurally colored surfaces with thin-film interferences and multiple reflections has been achieved on the basis of optical calculations^25–28^. Not only the one-dimensional structural colors of the layered structures but also a three-dimensional structural color of a morpho butterfly has also been visualized by three-dimensional modeling numerical simulations^29,30^. This realistic rendering is expected to be used as a tool for designing industrial products with such structural colors^31^.

In pearl grading, the value of a pearl is determined by several factors such as size, color, and luster among others^32–38^. To evaluate pearl quality according to these factors, regression analysis has been proposed for Akoya pearl grading^35^. Although the pearl grading includes many complex factors, pink pearls generally tend to be prefer. To control the color of cultured pearls, we have introduced nondestructive method of measuring the average aragonite crystal layer thickness in Akoya pearl farming^38^. This nondestructive method allows us to roughly control the color of cultured Akoya pearls. On the other hand, it is not completely clear what a pearl with a certain aragonite crystal layer thickness will look like. Since it takes at least one year to produce pearls in pearl farming, visual simulation of pearls based on the layer thickness is important to improve the productivity of pearls with the desired color. Some studies on the visual simulation of Akoya pearls have achieved a realistic computer graphics rendering^39,40^. In those studies, the interference color of Akoya pearls is determined from only the reflection, and then the texture and reflection intensity are adjusted to produce images of pearls. However, Raman mentioned that the reflection at a spherical surface is not sufficient to visualize the appearance of pearls and the beauty of pearls is due to the superposition of the reflected light and chromatic diffusion halo^8,9^. He considered that the halo is produced by transmission diffraction from the individual crystallites in aragonite layers in which each crystallite functions as a diffracting particle. Komatsu has also pointed out that pearls have reflection and transmission interference colors in which the transmission interference color is complementary to the reflection interference color^36,37^. According to his book, the color of Akoya pearls we see is the transmission interference color^37^. However, the mechanism is not fully understood yet. The important point here is that both Raman and Komatsu studied the appearance of pearls by focusing on the transmitted light from pearls. In general, the appearance of structural colors is determined by the reflected light, but in the case of pearls, it is interesting that the transmitted light is more important than the reflected light. Therefore, we have developed a new optical model appropriate for pearls based on light transmission, reflection, and scattering in pearls. The transmission and reflection spectra of Akoya pearls of three different colors calculated using the proposed model are in good agreement with the measured spectra. We have also developed a rendering program coded using OpenGL Shading Language to reproduce the appearance of pearls using the calculated optical spectra. The rendered images have high reproducibility of the interference colors of actual pearls. Furthermore, the appearance of pearls with different aragonite crystal layer thicknesses is predicted by the calculation based on the optical model.

## Results

**Figure 1:**
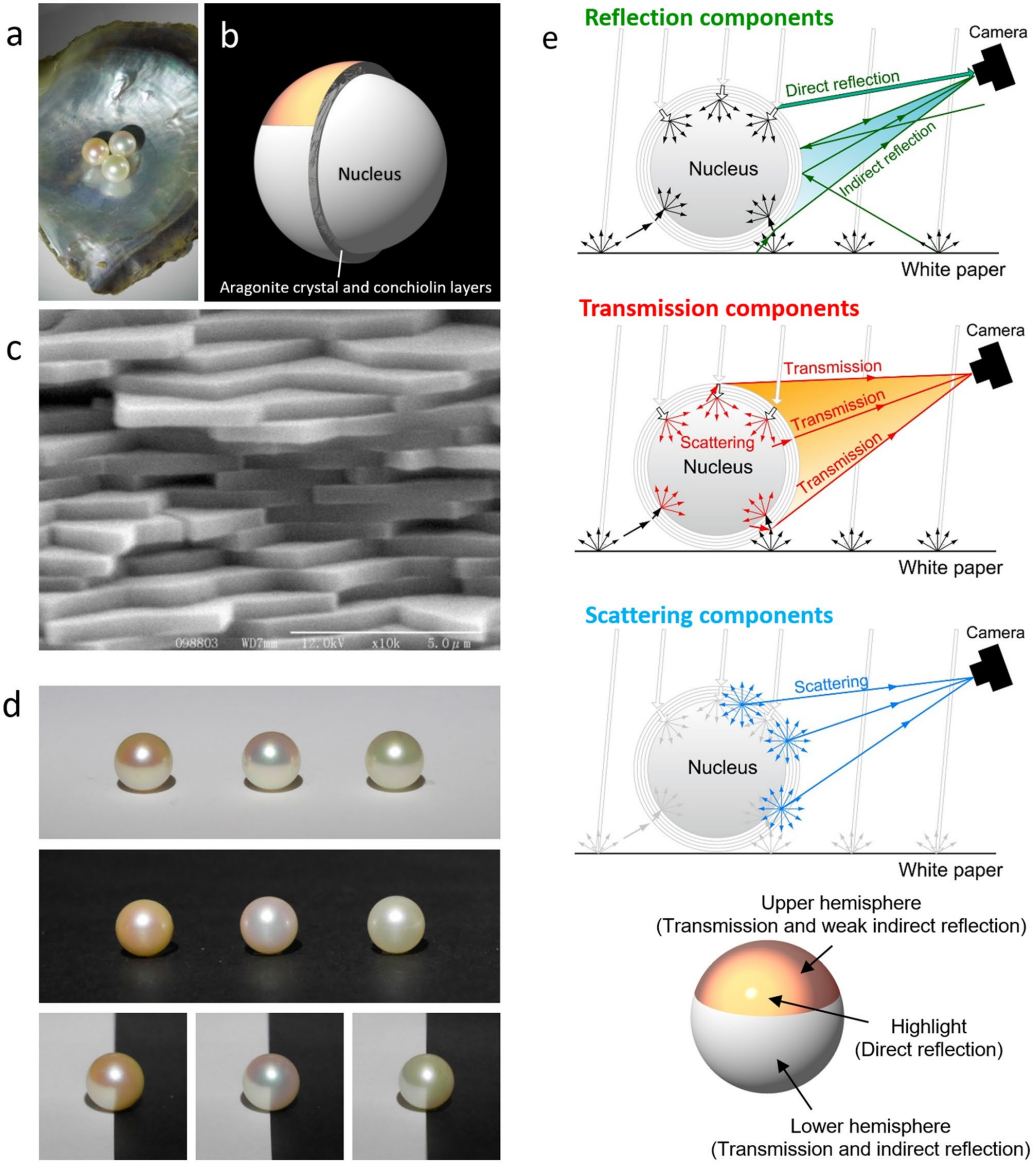
Structure, appearance, and optical model of pearls. (**a**) Three Akoya pearls of different colors. (**b**) Schematic illustration of structure of Akoya pearl. (**c**) SEM image of typical nacre of Akoya pearl. The length of the scale bar is 5 μm. (**d**) Photographs of three pearls placed on white and black sheets of paper. (**e**) Optical model for reflection, transmission, and scattering components of pearl.

Pearls show various beautiful structural colors, which depend on the layer thickness in nacre (Fig. 1a). The nucleus of a pearl is covered with nacre, which consists of aragonite crystal layers and conchiolin sheets (Fig. 1b). These multilayers in nacre can be clearly observed by a scanning electron microscopy (Fig. 1c). The thickness of an aragonite crystal layer is typically between 300 and 500 nm, and that of a conchiolin sheet is about 10 nm. In this study, we used three different colors of Akoya pearls (Fig. 1d) to investigate the mechanism underlying the structural color of pearls. The average thicknesses of the aragonite crystal layers of the orange, pink-green, and green pearls were 320, 360, and 410 nm, respectively, which were determined from their reflection spectra.

The appearance of pearls is affected by surrounding conditions. The effect of the surrounding conditions is clear when the pearls are placed against white and black background (Fig. 1d). Against the white background, the upper hemispheres of the three pearls show different colors, whereas their lower hemispheres all appear white. In contrast, the structural colors appear on almost the entire surface of the pearls against the black background. The difference is more clearly seen by placing it at the center of the white and black sheets of paper. These pictures were taken in a dark room to prevent the projection of the environment onto the surface of the pearls, and only one white LED was used for lighting (Supplementary Fig. 1). To explain these observations, we propose a new optical model for pearls (Fig. 1e). Reflection components detected by a camera consist of direct and indirect reflections. In the schematic illustration of the reflection components, white arrows represent light from the light source and green arrows represent the reflection components detected by the camera. That is, the camera captures the direct reflection from the light source at a point in the upper hemisphere and the indirect reflection from the white paper, which mainly illuminates the lower hemisphere of the pearl. In this case, the indirectly reflected light is much weaker than the directly reflected light from the light source. In the schematic illustration of the transmission components, the red arrows represent the transmission components arising from light scattering in the nucleus. In general, nucleuses are made of freshwater mussel and look like white beads (Supplementary Fig. 2). Nucleuses have a layered structure with a layer thickness of several microns. Light in a pearl is scattered at boundaries of layered structures in nacre and nucleus due to nonuniformity of the layered structures. The light scattering plays an important role in the appearance of pearls. Since the light from the light source does not enter into the camera directly under the lighting conditions (Supplementary Fig. 1), the detected transmission light is the scattered light. That is, the light coming from various directions is scattered in the nacre and nucleus, and then the light passes through the nacre and out into the air. In other words, the nucleus functions similarly to a light source. Since the scattered light passes through the nacre consisting of multilayers, transmission intensities of the light in certain wavelength regions decrease owing to multiple reflections in the nacre. For this reason, the transmitted light comes from not only the upper hemisphere but also the lower hemisphere. Therefore, the transmission interference colors can be seen from the entire surface of the pearl on the black paper in which reflection is suppressed (Fig. 1d). On the other hand, the transmission interference color is complementary to the reflection interference color. The observer will see the superposition of the transmission and reflection interference colors at the lower hemisphere. The two overlapping complementary colors produce the white color on the lower hemisphere when the pearl is placed on the white paper. Interestingly, according to the model, the color of the upper hemisphere is determined by the transmitted light, even though the pearl is irradiated with light from above (Supplementary Fig. 1). We have here explained that the transmission interference color is complementary to the reflection interference color, but strictly speaking, these are not completely complementary owing to light absorption of conchiolin.

**Figure 2:**
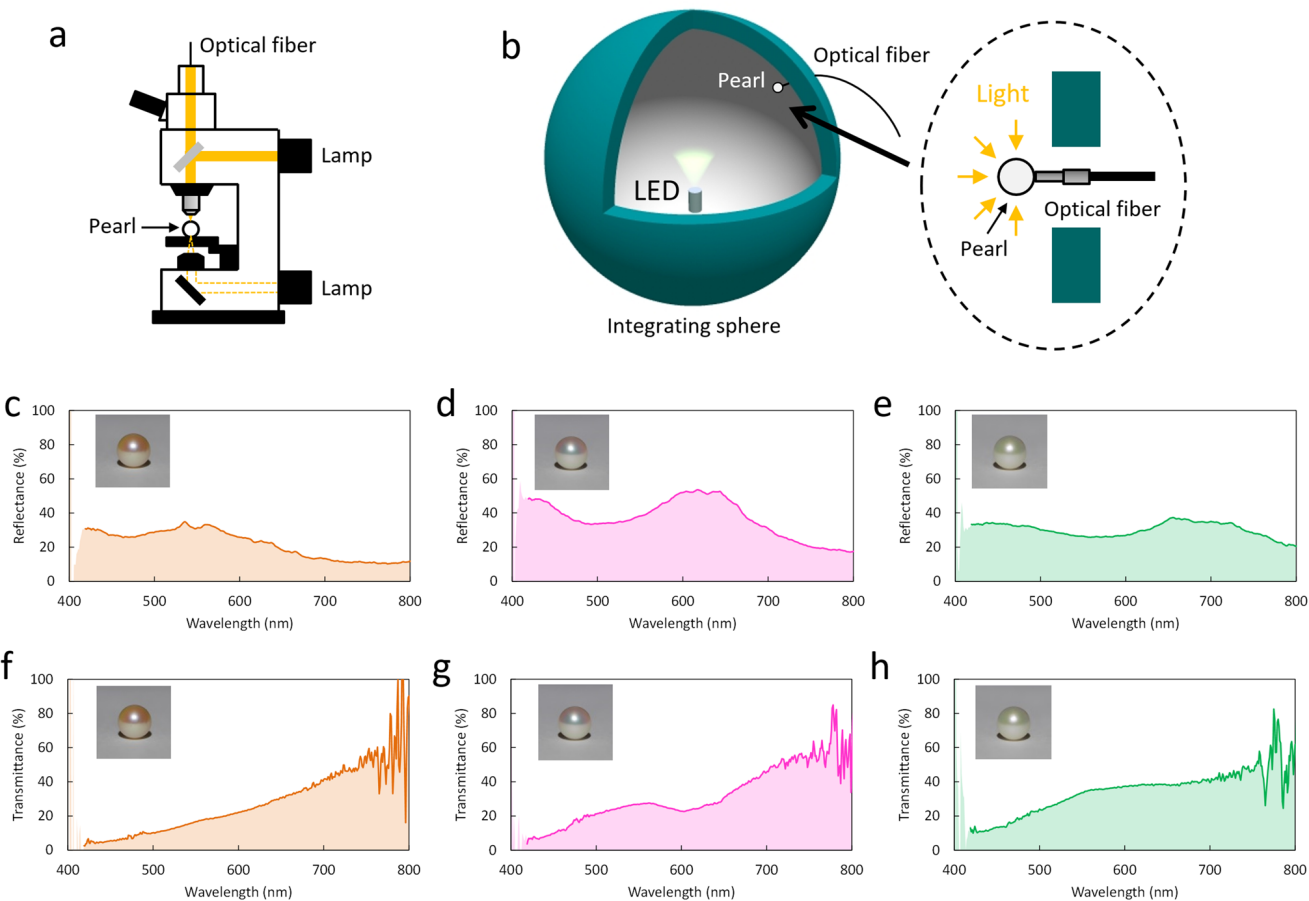
Reflection and transmission characteristics of pearls of three different colors. (**a**) Schematic illustration of microspectroscopy setup for reflection measurement. (**b**) Schematic illustration of experimental setup for transmission measurement using integrating sphere. (**c**–**e**) Reflection spectra of orange, pink-green, and green pearls, respectively. (**f**–**h**) Transmission spectra of the three pearls.

To investigate their optical properties, the transmission and reflection spectra of the three pearls were measured. The reflection spectra were obtained by microspectroscopy (Fig. 2a), which can reduce the nonuniformity of reflection wavelengths depending on the position because of the small observation area. In contrast, the transmission spectra cannot be measured by microspectroscopy because the pearls scatter light and the optical path is long. To obtain transmission properties, we have developed a measurement method for a transmission spectrum of a pearl using an integrating sphere, in which incident light from an LED enters the entire surface of a pearl except for the detector position (Fig. 2b). We consider that the system is suitable for measuring the transmission characteristics of pearls and verifying of the proposed transmission model (Fig. 1e). In the reflection spectra of the orange, pink-green, and green pearls (Fig. 2c–e), broad reflection bands show peaks corresponding to the center wavelength of multiple reflections from the aragonite crystal layers in nacre. The center wavelength *λ*_m_ of the reflection band for a normal incidence is given by *λ*_m_ = 2(*n*_1_*d*_1_ + *n*_2_*d*_2_)/*m*, where *n*_1_ and *n*_2_ are the refractive indices of the aragonite crystal and conchiolin layers, *d*_1_ and *d*_2_ are the thicknesses of the aragonite crystal and conchiolin layers, and *m* is the order of reflection. The measured center wavelengths of the 2nd-order reflection bands for the orange, pink-green, and green pearls were approximately 560, 620, and 700 nm, respectively. The thickness of the aragonite crystal layer *d*_1_ in nacre was determined using the above equation. The aragonite crystal layer thicknesses of the orange, pink-green, and green pearls were estimated as 324, 360, and 408 nm, respectively, where we used the following parameters: *n*_1_ = 1.68, *n*_2_ = 1.5, and *d*_2_ = 10 nm. The three spectra also indicate that light is scattered in the pearls because the baseline is not zero and the light at shorter wavelengths is scattered more than that at longer wavelengths. In the transmission spectra of the three pearls (Fig. 2f–h), the light scattering reduces the transmittance on the short-wavelength side, which corresponds to the reflection spectra. Furthermore, it can be seen that the stop bands of the pearls correspond to the reflection bands in the reflection spectra. Although the stop band of the orange pearl is very weak, we consider that the absorption of conchiolin in the orange pearl is stronger than those in the other two pearls.

**Figure 3:**
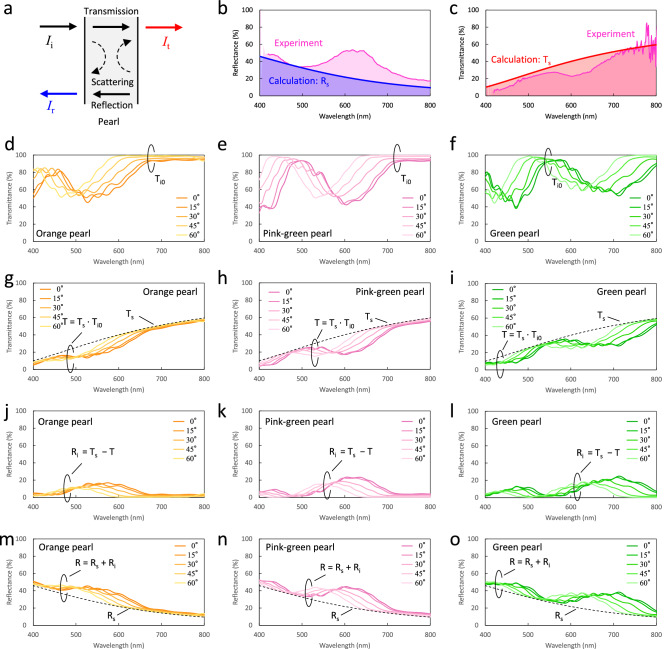
Calculated scattering and interference spectra of three pearls. (**a**) Schematic illustration of approximate model of scattering in pearl. (**b**) Calculated scattering reflection spectrum *R*_s_ and measured reflection spectrum of pink-green pearl. (**c**) Calculated scattering transmission spectrum *T*_s_ and measured transmission spectrum of pink-green pearl. (**d**–**f**) Calculated interference transmission spectra without scattering *T*_i0_ of orange, pink-green, and green pearls, respectively. The incident light is P-polarized and the legend represents an incident angle. (**g**–**i**) Calculated transmission spectra *T* of pearls obtained by *T* = *T*_s_ ∙ *T*_i0_ . (**j**–**l**) Calculated interference reflection spectra *R*i of pearls. (**m**–**o**) Calculated reflection spectra *R* of pearls obtained by *R* = *R*_s_ + *R*_i_.

To theoretically reproduce the transmission and reflection spectra, we have first calculated the spectral baselines on the basis of the Kubelka–Munk theory, which is a classical approximation for multiple scatterings^41,42^. In this calculation, the light scattering in the pearls was calculated by assuming that the pearls are a scattering medium and by simplifying the input and output of light (Fig. 3a). In actual pearls, the behavior of the light transmission and reflection is three dimensional, but we used the one-dimensional model to calculate their scattering. Although the model includes the assumption, the calculated scattering reflection *R*_s_ and transmission *T*_s_ are in good agreement with the baselines of the measured spectra (Fig. 3b,c).

**Figure 4:**
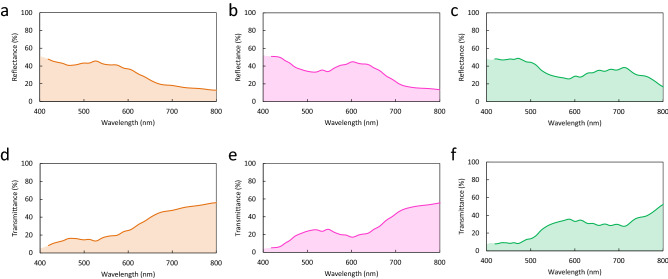
Calculated reflection and transmission spectra of three pearls. (**a**–**c**) Calculated reflection spectra of orange, pink-green, and green pearls, respectively. The incident angle is 0°. (**d**–**f**) Calculated transmission spectra of three pearls for normal incidence.

Interference components arising from the multiple reflections in nacre were calculated by the transfer matrix method, which is widely used for the analysis of a one-dimensional layered structure^43^. To calculate transmission and reflection spectra of pearls, the determination of the thickness distribution of aragonite crystal layers is necessary^44^. In general, the thickness of an aragonite crystal layer in nacre gradually decreases as a pearl grows^45^. To reproduce the characteristics of nacre, aragonite crystal layer thickness profiles were numerically generated by random decrement technique (Supplementary Fig. 3a–c). That is, the aragonite crystal layer is thick near the nucleus and gradually and randomly thins toward the surface. In this calculation, the number of aragonite crystal layers was set to be 1000, and the thickness was assumed to exhibit a normal distribution (Supplementary Fig. 3d–f). Assuming the distributions for the orange, pink-green, and green pearls, the transmittance without light scattering *T*_i0_ can be calculated by the transfer matrix method (Fig. 3d–f). The transmission spectra including light scattering in the pearls can be reproduced by combining the scattering and interference transmission calculations. Since the scattered light in the nucleus passes through the aragonite crystal layers, the transmission spectrum of the pearls *T* is given by *T*_s_∙*T*_i0_ (Fig. 3g–i). In other words, the scattered light in the nucleus acts as a light source and the aragonite crystal layers function as a transmission color filter. In addition, using the result of the transmission calculation, we can obtain the interference reflection component *R*_i_ by *T*_s_ − *T* (Fig. 3j–l). Since the reflection from the pearls is a superposition of the scattering reflection *R*_s_ and interference reflection *R*_i_, the reflection spectrum *R* is given by *R*_s_ + *R*_i_ (Fig. 3m–o). The reason why the interference reflection *R*_i_ decreases at shorter wavelengths is that the scattering reflection *R*_s_ increases at the shorter wavelengths. On the basis of the above procedure, we calculated the reflection and transmission spectra for P- and S-polarizations including the light scattering of the pearls. The calculated reflection and transmission spectra for the orange, pink-green, and green pearls at normal incidence (Fig. 4a–f) show good agreement with the experimental results (Fig. 2c–h).

**Figure 5:**
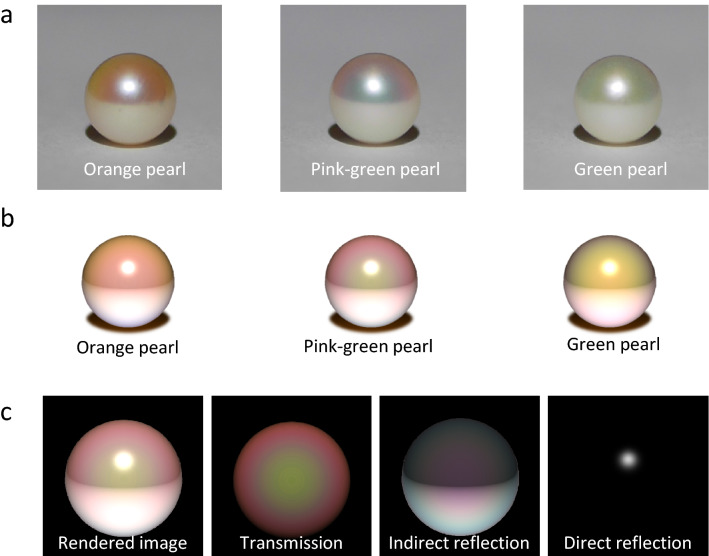
Real pearls and rendered images. (**a**) Photographs of three pearls of different colors. (**b**) Rendered images of pearls obtained using proposed optical model. (**c**) Transmission, indirect reflection, and direct reflection components of rendered image of pink-green pearl.

The visual simulator for the pearls has been coded using OpenGL Shading Language. Using the self-coding shading program and the calculated reflection and transmission spectra, we reproduced the appearance of the pearls with different aragonite crystal layer thicknesses. The appearance of the simulated pearls is in excellent agreement with that of real pearls (Fig. 5a,b). For example, in the real and rendered images of the green pearl, most of the upper hemisphere is green. In the real and rendered images of the orange pearl, the color of the upper hemisphere changes from dark orange to light orange with increasing distance from the center. Similarly, in the real and rendered images of the pink-green pearl, the color of the upper hemisphere gradually changes from green to pink. Furthermore, the lower hemisphere in all real and rendered images of the pearls is white. We have also rendered the image with a different viewing position (Supplementary Fig. 4). The simulation results are in good agreement with the real images, even at a different angle. These indicate that our models and optical calculations are appropriate for pearls.

The rendered images consist of three color components: transmission and reflection interference colors and a highlight (Fig. 5c). In the transmission component, the pearl has the transmission interference color depending on the angle. We here assumed that the light scattering in the nucleus and aragonite crystal layers is isotropic light scattering and that the intensities of the light scattered in all directions are homogeneous. The color of the transmission light is determined by the multiple interferences in the aragonite crystal layers. The light from the center of the pearl to an observer is incident at normal incidence, and the incident angle for the observer increases with the distance from the center (Supplementary Fig. 5). The calculated transmission spectrum at normal incidence (Fig. 4e) has two broad peaks at around 530 and 800 nm, in which the color of the peak at 530 nm corresponds to green and the peak at 800 nm does not strongly affect the visualization owing to infrared light. When the incident angle increases, the spectrum shifts to shorter wavelengths (Fig. 3d–o). At highly oblique incidence, the shorter broad peak shifts to the blue wavelength region and the longer broad peak shifts to the red wavelength region. Therefore, the color of the pearl gradually changes from green to pink. Furthermore, the transmission image is similar to that of the pearls placed on the black paper (Fig. 1d), in which the reflection light is suppressed by the black paper. On the other hand, the indirect reflection components strongly depend on the surrounding conditions. We here calculated the reflection interference color by irradiating the pearl with light indirectly reflected from the white paper (Fig. 1e). In this situation, the light from the white paper mainly brightens the lower hemisphere of the pearl, and the reflection interference color also depends on the angle and position. Note that reflection interference colors are complementary to transmission interference colors. That is, from the comparison between the transmission and indirect reflection images, the green and pink parts are opposite to each other. This is because the peak and dip wavelengths are opposite between the transmission and reflection interference colors (Fig. 4). The highlight of the direct reflection from the light source is assumed to be white owing to the light saturation of the detector. The rendered image is the superposition of all images that considers the scattering, interference, indirect reflection, and direct reflection of light. Now, we can clearly explain that the lower hemisphere of the pearl on the white paper is white. This is because the lower hemisphere color is due to the superposition of the transmission interference and complementary reflection interference colors.

**Figure 6:**
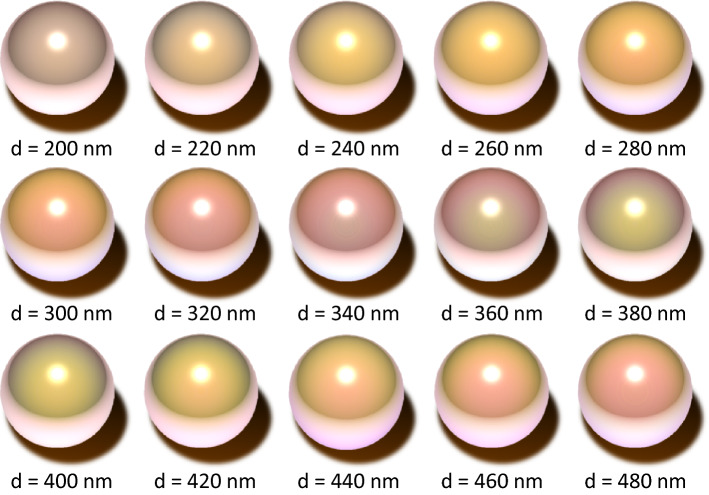
Simulation of appearance of Akoya pearls. The average aragonite crystal layer thickness *d* gradually changes. The variance *σ*^2^ of all layer thickness distributions is set to be *σ*^2^ = 30 nm.

The most important application of the simulation is the visualization of pearls, which depends on the layer structure in nacre. The images of pearls have been rendered with different average aragonite crystal layer thicknesses (Fig. 6). The pearl with *d* = 200 nm is green, which gradually changes to yellow, orange, pink-green, green, and yellowish-orange with increasing layer thickness. This is the most important result of the present study. These images suggest that the color of pearls changes repeatedly from green to orange and pink-green. Furthermore, interestingly, the range of conditions for making pink-green pearls, which are more expensive than those of other colors in the market, is narrow. The prediction of color patterns in pearls would greatly help pearl researchers and farmers academically and practically.

## Discussion

The optical properties of pearls have been investigated by observing changes in appearance depending on the surrounding environment and measuring reflection and transmission spectra. The reflection spectra of pearls were measured by conventional microspectroscopy, whereas the transmission spectra of pearls could not be measured owing to a strong scattering. To measure the transmission properties of the pearls, an integrating sphere was used for the measurement of transmission spectra. On the other hand, to explain the changes in appearance depending on the surrounding environment, we have developed an optical model that considers the scattering in pearls and the transmission and reflection interferences in nacre. The scattering in the nucleus and nacre has been calculated on the basis of the Kubelka–Munk theory, and the multiple interference colors in nacre have been calculated by the transfer matrix method. The transmission and reflection spectra calculated using the proposed model are in good agreement with the experimental results. We have also developed the pearl visual simulator coded by OpenGL Shading Language. By using the simulator and the calculated spectra, we have succeeded in visualizing Akoya pearls with different interference colors on a computer. We hope that our proposed model becomes the new standard for analyzing the optical properties of pearls.

## Methods

### Spectral measurements

The reflection spectra of the three pearls were measured by microspectroscopy. A pearl on the stage was irradiated with a halogen coaxial epi-illuminator. The reflected light was detected with an optical fiber connected to a CCD multichannel spectrometer (StellarNet, BLUE-Wave). The microspectroscopy can reduce the error due to the nonuniformity of reflection wavelengths because of the small observation area. In contrast, transmission spectra cannot be measured by microspectroscopy because pearls scatter light and the optical path is long. To obtain effective transmission properties, we measured the transmission spectra of the pearls using an integrating sphere. As shown in Fig. 2b, a pearl attached to an optical fiber was placed in the integrating sphere, to which the light enters the entered and illuminated surface of the pearl except for the detector position. A white LED was used as a light source and the light intensity with and without the pearl was measured with the spectrometer.

### Calculations of transmission and reflection spectra

Light scattering in a pearl was calculated on the basis of the Kubelka–Munk theory, which is widely used to analyze multiple light scatterings. Transmission and reflection intensities through a multiple-scattering medium are expressed as

1 $${T}_{\mathrm{s}}=\frac{\left(1-{A}^{2}\right)\mathrm{exp}(-\alpha \tau )}{1-{A}^{2}\mathrm{exp}(-2\alpha \tau )},$$

2 $${R}_{\mathrm{s}}=\frac{1-\mathrm{exp}(-2\alpha \tau )}{1-{A}^{2}\mathrm{exp}(-2\alpha \tau )},$$

where *τ* is the optical length of the scattering medium, and *A* and *α* are given by

3 $$\alpha =\sqrt{K\left(K+2S\right)},$$

4 $$A=\frac{K+2S-\alpha }{K+2S+\alpha }.$$

Here, *K* and *S* are nondimensional absorption and scattering coefficients of the scattering medium, respectively. In this study, the coefficients are set to be *K* = (300 × 10^−9^/*λ*)/τ and *S* = [(500 × 10^−9^/*λ*)^4^]/τ. It is assumed that the pearl has a constant extinction coefficient and Rayleigh scattering occurs in the pearl.

To obtain interference components, the transmission spectrum *T*_i0_ of the pearl without scattering was calculated by the transfer matrix method, which is widely used for the analysis of a one-dimensional layered structure. Since the light transmitted from the pearl passes through the multilayer and scattering medium in series, the total transmission spectrum *T* including light scattering was calculated as *T* = *T*_s_∙*T*_i0_. On the other hand, since one observes the superposition of the scattering reflection *R*_s_ and interference reflection *R*_i_, the total reflection spectrum *R* including light scattering was calculated as *R* = *R*_s_ + *R*_i_. In the interference calculations, we used the following parameters: the thickness of the conchiolin is constant at 10 nm and the refractive indices of the aragonite crystal and conchiolin are 1.68 and 1.5, respectively. Pearls have not only the aragonite layer thickness nonuniformity (Supplementary Fig. 3) but also an in-plane nonuniformity. In the calculations, a Gaussian filter is applied to reproduce the in-plane nonuniformity of pearls.

### Rendering of pearl

The pearl visualization simulator has been coded using the OpenGL Shading Language, in which the colors of pearls are determined by optical calculations. The colors of pearls consist of transmission, direct reflection, and indirect reflection components. The transmission interference color has angular dependence. However, the transmission color does not depend on the observer position because the nucleus functions as a light source. Since the transmission color changes depending on the distance from the center under the condition, the transmission components of the pearl are concentrically colored in the program. The color of the direct reflection is set to be white because the intensity of the direct reflection is very high. The indirect reflection from the white paper is reproduced by placing a pseudo light source under the pearl. The reflection color is complementary to the transmission color. Unlike transmission, the light intensity of reflection depends on the position of the viewpoint. In addition, the intensities of the light source for transmission and reflection differ in the program. This is due to the fact that the transmission light depends on the actual light source, whereas the indirect reflection depends on the surrounding environment.

## Supplementary Information

Supplementary Figures.

## References

[1] Brewster, D. *A Treatise on Optics* (Carey, Lea, and Blanchard, 1833).

[2] Pfund AH (1917). The colors of mother-of-pearl. J. Franklin Inst..

[3] Rayleigh L (1923). Studies of iridescent colour and the structures producing it. II Mother-of-pearl. Proc. R. Soc. Lond. A.

[4] Raman CV (1935). On iridescent shells. Part I. Introductory. Proc. Indian Acad. Sci..

[5] Raman CV (1935). On iridescent shells. Part II. Colours of laminar diffraction. Proc. Indian Acad. Sci..

[6] Raman CV, Krishnamurti D (1954). The structure and optical behaviour of iridescent shells. Proc. Indian Acad. Sci..

[7] Raman CV, Krishnamurti D (1954). The structure and optical behaviour of pearls. Proc. Indian Acad. Sci..

[8] Raman CV, Krishnamurti D (1954). On the chromatic diffusion halo and other optical effects exhibited by pearls. Proc. Indian Acad. Sci..

[9] Raman CV, Krishnamurti D (1954). Optics of the pearl. Curr. Sci..

[10] Kinoshita S (2011). Deep sequencing of ESTs from nacreous and prismatic layer producing tissues and a screen for novel shell formation-related genes in the pearl oyster. PLoS One.

[11] Takeuchi T (2012). Draft genome of the pearl oyster *Pinctada fucata*: A platform for understanding bivalve biology. DNA Res..

[12] Funabara D (2014). Novel genes participating in the formation of prismatic and nacreous layers in the pearl oyster as revealed by their tissue distribution and RNA interference knockdown. PLoS One.

[13] Takeuchi T (2020). Divergent northern and southern populations and demographic history of the pearl oyster in the western Pacific revealed with genomic SNPs. Evol. Appl..

[14] Rayleigh L (1917). On the reflection of light from a regularly stratified medium. Proc. R. Soc. Lond. A.

[15] Parker AR (2000). 515 million years of structural colour. J. Opt. A Pure Appl. Opt..

[16] Vukusic P, Sambles JR (2003). Photonic structures in biology. Nature.

[17] Kinoshita S, Yoshioka S (2005). Structural colors in nature: The role of regularity and irregularity in the structure. ChemPhysChem.

[18] Sun J, Bhushan B, Tong J (2013). Structural coloration in nature. RSC Adv..

[19] Kimura T (2020). Guanine crystals regulated by chitin-based honeycomb frameworks for tunable structural colors of sapphirinid copepod, *Sapphirina nigromaculata*. Sci. Rep..

[20] Barrera-Patiño CP (2020). Photonic effects in natural nanostructures on *Morpho cypris* and *Greta oto* butterfly wings. Sci. Rep..

[21] Joannopoulos JD, Villeneuve PR, Fan S (1997). Photonic crystals: Putting a new twist on light. Nature.

[22] Sakoda, K. *Optical Properties of Photonic Crystals* (Springer, 2001).

[23] Ozaki R (2018). Luminescent color control of Langmuir–Blodgett film by emission enhancement using a planar metal layer. Sci. Rep..

[24] Lee K-T (2019). Flexible high-color-purity structural color filters based on a higher-order optical resonance suppression. Sci. Rep..

[25] Sun, Y., Fracchia, F. D., Drew, M. S. & Calvert, T. W. Rendering iridescent colors of optical disks. In *EGSR 2000: Rendering Techniques 2000* 341–352 (2000).

[26] Hirayama H, Kaneda K, Yamashita H, Monden Y (2001). An accurate illumination model for objects coated with multilayer films. Comput. Graph..

[27] Iwasaki K, Matsuzawa K, Nishita T (2004). Real-time rendering of soap bubbles taking into account light interference. Proc. Comput. Graph. Int..

[28] Wu FK, Zheng CW (2015). Microfacet-based interference simulation for multilayer films. Graph. Models.

[29] Okada N (2013). Rendering Morpho butterflies based on high accuracy nano-optical simulation. J. Opt..

[30] Musbach A, Meyer GW, Reitich F, Oh SH (2013). Full wave modelling of light propagation and reflection. Comput. Graph. Forum.

[31] Ershov S, Kolchin K, Myszkowski K (2001). Rendering pearlescent appearance based on paint-composition modelling. Comput. Graph. Forum.

[32] Elen S (2001). Spectral reflectance and fluorescence characteristics of natural-color and heat-treated "Golden" south sea cultured pearls. Gems Gemol..

[33] Snow MR, Pring A, Self P, Losic D, Shapter J (2004). The origin of the color of pearls in iridescence from nano-composite structures of the nacre. Am. Mineral..

[34] Toyota T, Nakauchi S (2013). Optical measurement of interference color of pearls and its relation to subjective quality. Opt. Rev..

[35] Tani Y, Nagai T, Koida K, Kitazaki M, Nakauchi S (2014). Experts and novices use the same factors—but differently—to evaluate pearl quality. PLoS One.

[36] Komatsu H, Yazaki J, Yamamoto T, Tanaka H, Yokose C (2014). Study of the measurement of Teri in pearls I: Consideration about the correlation of three kinds of evaluation methods of Teri in white Akoya pearls. J. Gemmol. Soc. Jpn..

[37] Komatsu, H. *Dictionary of Pearls* [Translated from Japanese.] (Senken shinbun, 2015) (**in Japanese**).

[38] Odawara K, Ozaki R, Takagi M (2017). Influence of the thickness of the nacreous elemental lamina of the pearl oyster *Pinctada fucata* used as donor oysters on the pearls. Nippon Suisan Gakkai Shi.

[39] Nagata N, Dobashi T, Manabe Y, Usami T, Inokuchi S (1997). Modeling and visualization for a pearl-quality evaluation simulator. IEEE Trans. Vis. Comput. Graph..

[40] Dobashi T, Nagata N, Manabe Y, Inokuchi S (1998). Implementation of a pearl visual simulator based on blurring and interference. IEEE ASME Trans. Mechatron..

[41] Kubelka P, Munk F (1931). Ein Beitrag zur Optik der Farbanstriche. Z. Tech. Phys..

[42] Ishibashi, A. *Wave Propagation and Scattering in Random Media* (IEEE Press-Oxford University Press, 1997).

[43] Born, M. & Wolf, E. *Principles of Optics: Electromagnetic Theory of Propagation, Interference and Diffraction of Light* (Pergamon Press, 1964).

[44] Ozaki R, Yoshimoto A, Watanabe G, Kadowaki K, Odawara K (2016). Calculation of reflection spectrum with actual layer thickness profile in nacre of akoya pearl oyster. J. Phys. Conf. Ser..

[45] Muhammad G, Atsumi T, Sunardi, Komaru A (2017). Nacre growth and thickness of Akoya pearls from Japanese and Hybrid *Pinctada fucata* in response to the aquaculture temperature condition in Ago Bay, Japan. Aquaculture.

