# Supplementary material for: Structural colors of pearls
Source: Sci Rep. 2021 Jul 27;11:15224. doi: 10.1038/s41598-021-94737-w (PMC8316437; doi:10.1038/s41598-021-94737-w)
Supplement: Supplementary file 1 — Supplementary Figures. [file 41598_2021_94737_MOESM1_ESM.pdf]

Supplementary Information

Structural colors of pearls

Ryotaro Ozaki[1,*], Kei Kikumoto[1], Masataka Takagaki[1], Kazunori Kadowaki[1] & Kazushi Odawara[2]

[1]Department of Electrical and Electronic Engineering and Computer Science, Graduate School of Science and Engineering, Ehime University, Matsuyama, 790-8577, Japan

[2]Fisheries Research Center, Ehime Research Institute of Agriculture, Forestry and Fisheries, 5516 Shimonada, Uwajima, Ehime 798-0104, Japan

*ozaki.ryotaro.mx@ehime-u.ac.jp

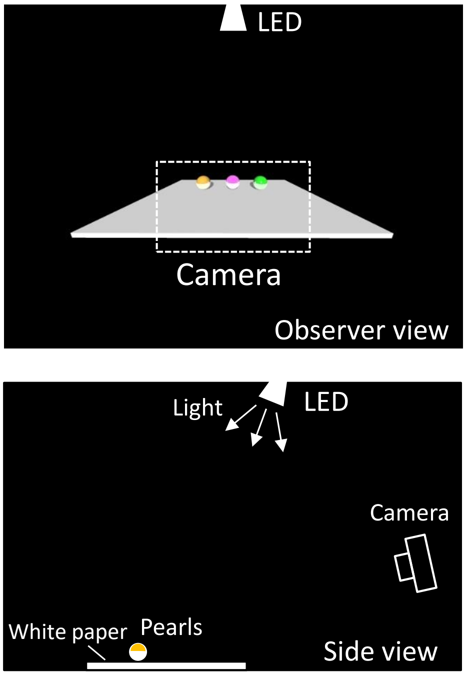

Supplementary Figure 1. a Observer view of dark room for taking photos of orange, pink-green, and green pearls. In the dark room, one LED is used to prevent complex lighting conditions. **b** Side view of dark room.

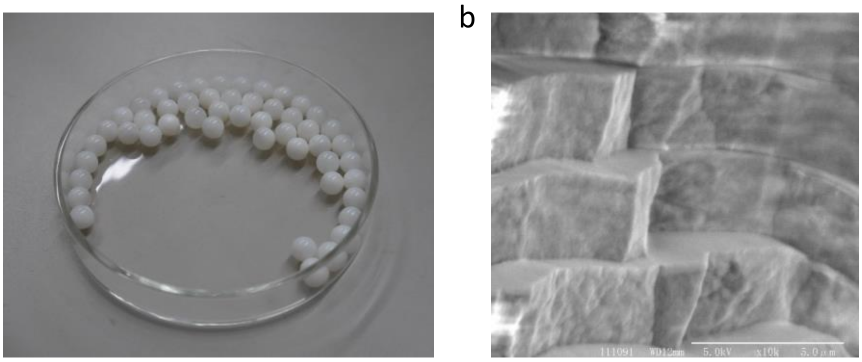

Supplementary Figure 2. a Nucleuses of pearls which are made of freshwater mussel. **b** SEM image of a typical nucleus of Akoya pearl. Nucleuses have a layered structure with a layer thickness of several microns. The length of the scale bar is 5 µm.

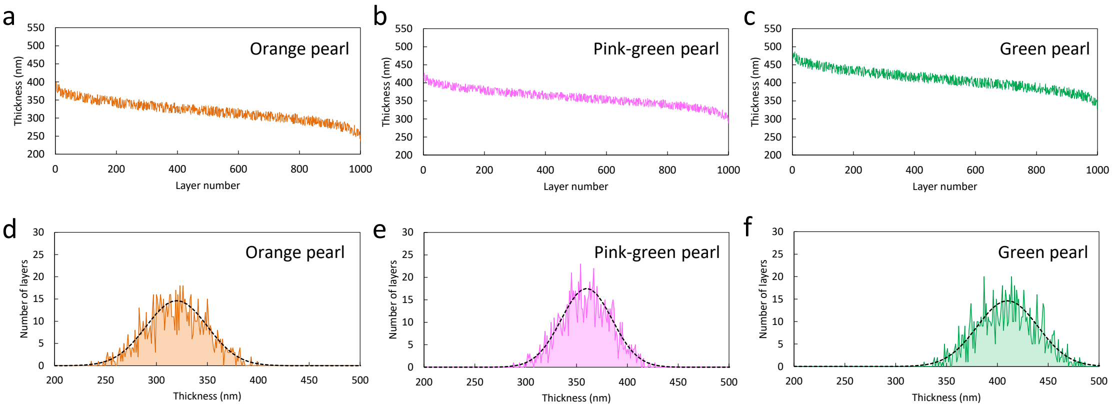

Supplementary Figure 3. a-c Aragonite crystal layer thickness profiles of orange, pink-green, and green pearls calculated using random decrement. In general, the aragonite crystal layer gradually thins from the nucleus to the surface. **d-e** Calculated layer thickness distributions of three pearls. The average thickness d and variance σ^2 of the distributions are as follows: (d) d = 320nm and σ^2 = 30 nm, (e) d = 360nm and σ^2 = 25 nm, and (f) d = 410nm and σ^2 = 30 nm.

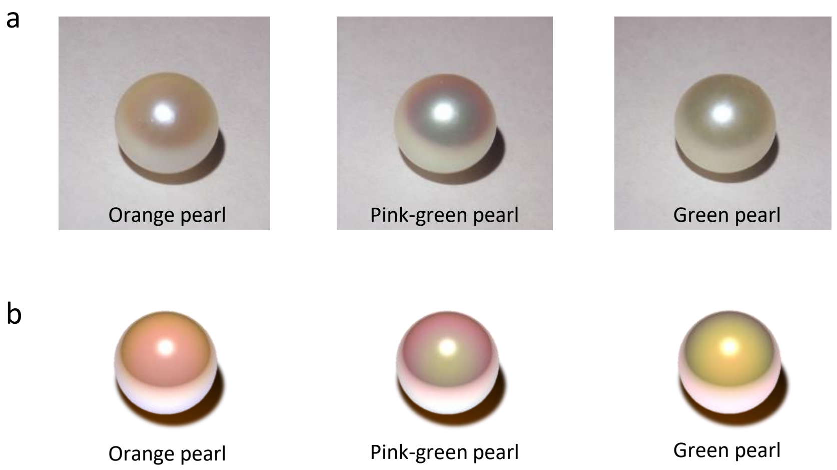

Supplementary Figure 4. a Photographs of three pearls of different colors from angle different from that in Fig. 5a. **b** Rendered images of pearls from different camera view.

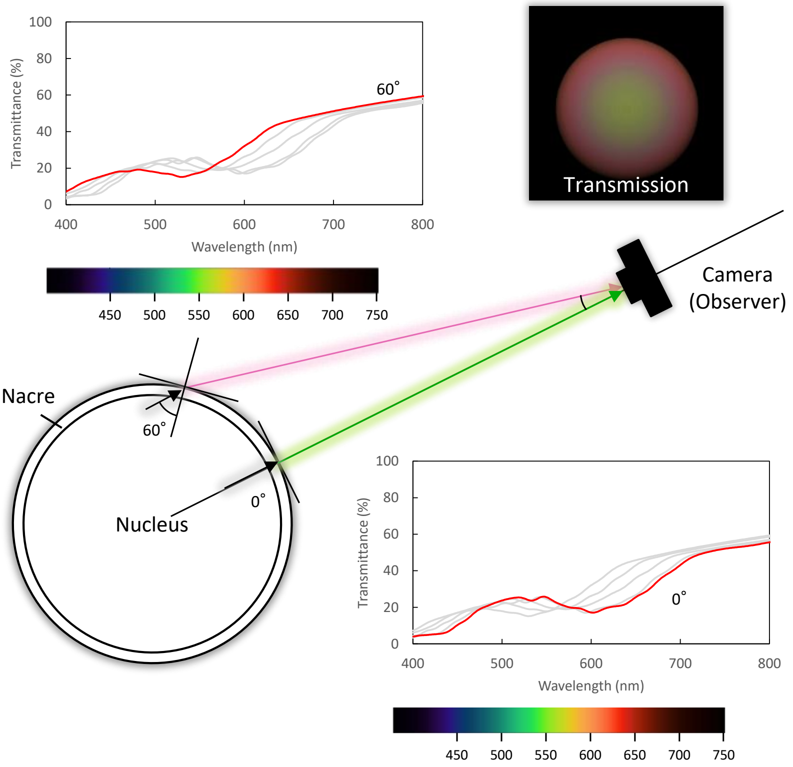

Supplementary Figure 5. Transmission interference color distribution and the mechanism. The light from the center of the pearl to an observer is incident at normal incidence, and the incident angle for the observer increases with the distance from the center. At normal incidence, the reflection peak appears at 530 nm, which corresponds to green. At highly oblique incidence, the shorter reflection peak shifts to the blue wavelength region and the longer broad peak shifts to the red wavelength region. Therefore, the color of the pearl gradually changes from green to pink.
